# P-1925. Comparison of Outcomes in Obese Versus Non-Obese Patients Receiving Fluconazole and/or Micafungin for the Treatment of Candidemia

**DOI:** 10.1093/ofid/ofaf695.2094

**Published:** 2026-01-11

**Authors:** Amber Welborn, Travis J Carlson, Gerard Gawrys, Amy Shum

**Affiliations:** University Health, San Antonio, Texas; The University of Texas at Austin College of Pharmacy, San Antonio, Texas; University Health, San Antonio, Texas; University Health, San Antonio, Texas

## Abstract

**Background:**

Clinical practice guidelines recommend initial echinocandin therapy followed by oral azole step-down therapy for the treatment of candidemia. However, the pharmacokinetics of these medications can be altered by patient characteristics, such as obesity. The purpose of this study was to compare outcomes in obese versus non-obese patients with candidemia treated with fluconazole and/or micafungin.

**Methods:**

This single-center, retrospective cohort study included adults with candidemia admitted from 1/1/20 - 12/1/24. Patients receiving < 48 hours of fluconazole and/or micafungin, those with resistance to both agents, or those not receiving antifungals within 24 hours of positive culture were excluded. Treatment failure, the primary composite outcome comprised of 14-day mortality, 14-day microbiologic failure, and 30-day candidemia recurrence, was compared between obese (BMI > 30 kg/m^2^) and non-obese patients. Secondary outcomes included hospital length of stay (LOS).

**Results:**

Ninety-six patients were included, 34 (35%) obese and 62 (65%) non-obese. Eleven (32%) obese and 7 (11%) non-obese patients received micafungin doses > 100 mg/day (P = 0.01). Among patients receiving fluconazole (n = 48), the median (interquartile range, IQR) weight-normalized dose to minimum inhibitory concentration (MIC) ratio was 6.6 (3.9-10.8) and 9.7 (3.3-21.2) in obese versus non-obese patients, respectively (P = 0.62). Treatment failure occurred in 13 (38%) obese and 20 (32%) non-obese patients (P=0.56). The median (IQR) LOS was 23 days in obese patients and 22 days in non-obese patients (P = 0.96).

**Conclusion:**

Obesity was not associated with treatment failure in this cohort of hospitalized patients with candidemia. Nearly one-third of obese patients received micafungin doses > 100 mg/day, and the weight-normalized fluconazole dose to MIC ratios did not significantly differ in obese versus non-obese patients. Depending on local epidemiologic factors, it may be prudent to consider higher doses of fluconazole and/or micafungin in obese patients with candidemia.

**Disclosures:**

Travis J. Carlson, PharmD, BCIDP, Aimmune Therapeutics, Inc.: Speaker bureau

